# Akt2 mediates glucocorticoid resistance in lymphoid malignancies through FoxO3a/Bim axis and serves as a direct target for resistance reversal

**DOI:** 10.1038/s41419-018-1043-6

**Published:** 2019-01-01

**Authors:** Mixue Xie, Apeng Yang, Jiexian Ma, Min Wu, Hongyue Xu, Kefei Wu, Youxin Jin, Yanhui Xie

**Affiliations:** 1 Department of Hematology & Shanghai Key Laboratory of Clinical Geriatric Medicine, Huadong Hospital Affiliated to Fudan University, Shanghai, 200040 China; 2 Senior Department of Hematology, The First Affiliated Hospital, College of Medicine, Zhejiang University, Hangzhou, Zhejiang 310003 China; 3 The School of Life Sciences, Shanghai University, Shanghai, 200444 China; 4 Department of Hematology & Rheumatology, The First Affiliated Hospital of Fujian Medical University, Fuzhou, Fujian 350005 China; 5 Department of Hematology & Oncology, The Second People’s Hospital of Liaocheng, Liaocheng, 252600 China

## Abstract

Glucocorticoids (GCs) are widely used drugs in the treatment of lymphoid malignancies; resistance of GCs in lymphocytes confers poor prognosis and the mechanisms are poorly understood. Here, we found T-acute lymphoblastic leukemia (T-ALL) cells acquire resistance to dexamethasone (DEX)-mediated killing through abnormal activation of Akt, resulting in inhibition of the FoxO3a/Bim pathway. The resistant state was reported to be associated with increased glycolysis, NOTCH1 activating mutations and activated PI3K/ serum GS regulated kinases (SGK) pathway. Use of aforementioned pathway inhibitors blocked FoxO3a-phosphorylation and partially improved DEX-mediated killing of GC-resistant T-ALL cells, further revealing the essential role of the FoxO3a/Bim pathway in the development of GC resistance. Inhibition of Akt is most effective at restoring sensitivity to DEX of GC-resistant lymphocytes in vitro and in vivo, but shows significant hepatotoxicity in vivo. A significantly elevated expression of Akt2 not Akt1 in intrinsically, secondarily GC-resistant lymphocytes and relapsed/refractory ALL patients implicates a more specific target for GC resistance. Mechanistically, Akt2 has a stronger binding capacity with FoxO3a compared to Akt1, and acts as a direct and major negative regulator of FoxO3a activity driving GC resistance. Pharmacologic inhibition of Akt2 more effectively restores sensitivity to GCs than inhibition of Akt1 in vitro, shows higher synergistic effect acting with DEX, and reverses GC resistance in GC-resistant T- or B- lymphoid tumors in vivo with reduced liver toxicity. In summary, these results suggest that Akt2 might serve as a more direct and specific kinase mediating GC resistance through FoxO3a/Bim signaling pathway, and Akt2 inhibition may be explored as a promising target for treating GC-resistant hematopoietic malignancies.

## Introduction

Glucocorticoids (GCs) are widely used drugs in the treatment of lymphoid tumors as a result of their ability to induce apoptosis in lymphoid progenitor cells. A major obstacle in GC therapy, however, is the gradual acquisition of apoptotic resistance in malignant hematopoietic cells repeatedly treated with these hormones. Previous reports indicate that between 15 and 30% of pediatric acute lymphoblastic leukemia (ALL) samples are resistant to GCs^1,2^, while in refractory childhood ALL, the prevalence of GC resistance is as high as 70%^3^. A poor response to prednisone after seven days of treatment is also a strong indicator of an increased risk of relapse and therapeutic failure in pediatric ALL^1,2^. Therefore, significant efforts are underway to develop novel strategies for resensitizing GC-resistant cells to GC therapy.

Mechanisms involved in GC resistance of hematopoietic tumors have yet to be elucidated, resulting in obstacles to the discovery of efficient approaches or treatments. Various FoxO transcription factors, especially FoxO3a, have been shown to regulate apoptosis in lymphocytes^4,5^. Indeed, the FoxO3a transcription factor is upregulated by GCs in 697 pre-B ALL cells^6^. Our previous study has also shown that FoxO3a plays an important role in GC-induced apoptosis of lymphocytes and sensitivity to dexamethasone (DEX) correlates negatively with expression of phosphorylated-(p-) FoxO3a^7^. A common mechanism of inactivation of FoxO transcription factors is directly phosphorylated by Akt^8^. Inhibition of Akt kinase with MK2206 enhances GC-induced apoptosis in T-ALL cell lines^9^. Grade 3 or 4 hematologic toxicities^10–12^ and common hepatic toxicities^10^ with increased aspartate aminotransferase (AST) and alanine aminotransferase (ALT) of Akt inhibitors have been reported in the treatment of solid tumors in humans, however, partially limit their clinical applicability.

There are two closely related, highly conserved homologs of Akt: Akt-1 and -2, each containing a PH region and a kinase domain^13–15^. There are obvious differences in enzyme function between Akt1 and Akt2. Akt1 is ubiquitously expressed and plays an important role in cell proliferation^16,17^ while Akt2 is expressed at high levels in skeletal muscle, in the β-islet cells of the pancreas and in brown fat and is involved in the regulation of blood sugar^16–18^. Fillmore et al.^19^ examined the expression of Akt1 and Akt2 in a variety of hematopoietic cell lines and found that the expression of Akt2 differed more than the expression of Akt1 in these hematopoietic cell lines. In human lens epithelial cells (HLECs) Akt2 is an essential kinase in counteracting oxidative-stress-induced apoptosis through promoting phosphorylation of FoxO3a and thus downregulating Bim expression^20^. The Akt2/FoxO3a/Bim pathway has been extensively studied in HLECs^20^. Therefore, in our current study, we examined the potential role of Akt isoforms Akt1 and Akt2 in the mechanism of GC resistance and explored an effective drug with less toxicity, as an option for treatment of GC-resistant hematopoietic malignancies.

## Results

### Aberrant activation of Akt/FoxO3a/Bim signaling pathway may be a mechanism of GC resistance in lymphoid tumor cells

**Fig. 1:**
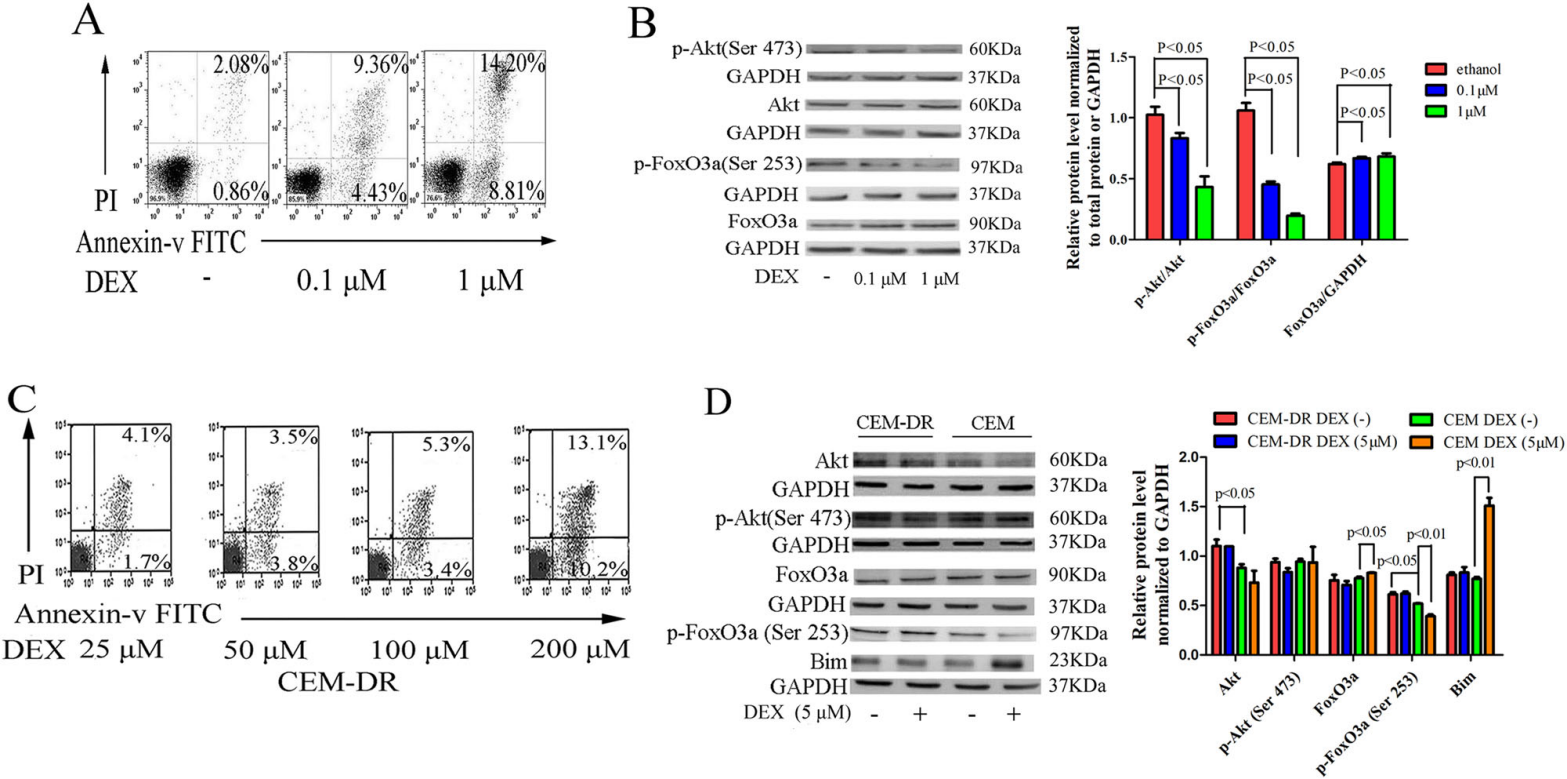
Aberrant activation of Akt pathway enhances the inhibitory effects on the FoxO3a/Bim, driving GC resistance. **a** Apoptotic cell death in cells treated with increasing concentrations of DEX (0 µM, 0.1 µM, 1 µM) as measured by flow cytometry. **b** Akt, p-Akt (Ser473), FoxO3a, and p-FoxO3a (Ser253) levels in CCRF-CEM cells treated with different concentrations of DEX (0, 0.1, 1 µM) were examined by western blot using the indicated antibodies. GAPDH was used as a loading control. **c** Analysis of apoptosis in CEM-DR cells following treatment with DEX (25, 50, 100, 200 µM) measured by flow cytometry. **d** Comparison of levels of Akt/FoxO3a/Bim signaling proteins in CCRF-CEM cells and CEM-DR cells with or without DEX treatment (5 µM) using western blot analysis. GAPDH was used as a loading control. Bar graphs in **b**, **d** represent mean ± SD

Unphosphorylated FoxO3a can be upregulated by DEX treatment and then translocate into nucleus and induce apoptosis in lymphocytes^7^. To examine the importance of the Akt/FoxO3a pathway in GC-induced apoptosis of lymphoid tumors we utilized CCRF-CEM cells, which are a moderately steroid-resistant cell line^21,22^. Increasing the concentration of DEX resulted in increased apoptosis of CCRF-CEM cells (Fig. 1a). Both the total p-Akt and p-FoxO3a levels, as well as the ratios of p-Akt (Ser473) to total Akt and p-FoxO3a (Ser253) to FoxO3a, decreased; the total FoxO3a expression increased (Fig. 1b). These results suggest that Akt is the major regulatory kinase that phosphorylates FoxO3a into an inactivated form and that upregulation of FoxO3a may be an indispensable process in GC-induced apoptosis. Treatment of CCRF-CEM cells with a specific concentration of DEX over multiple passages resulted in the generation of a highly resistant cell line, designated CEM-DR (Fig. 1c). In CEM-DR cell lines, the expressions of Akt and p-FoxO3a (Ser253) increased when compared with CCRF-CEM. Treatment of CCRF-CEM cells with DEX resulted in decreased p-FoxO3a (Ser253) expression, increased expressions of total FoxO3a and the pro-apoptotic protein Bim. While in CEM-DR cells, there were no significant changes observed in these proteins with a concentration of 5 µM of DEX treatment (Fig. 1d). This finding suggests that the aberrant activation of the Akt pathway may be one of the mechanisms of GC resistance in lymphocytes.

### Inhibition of Akt leads to a greater increase in sensitivity to GCs than inhibition of other pathway

**Fig. 2:**
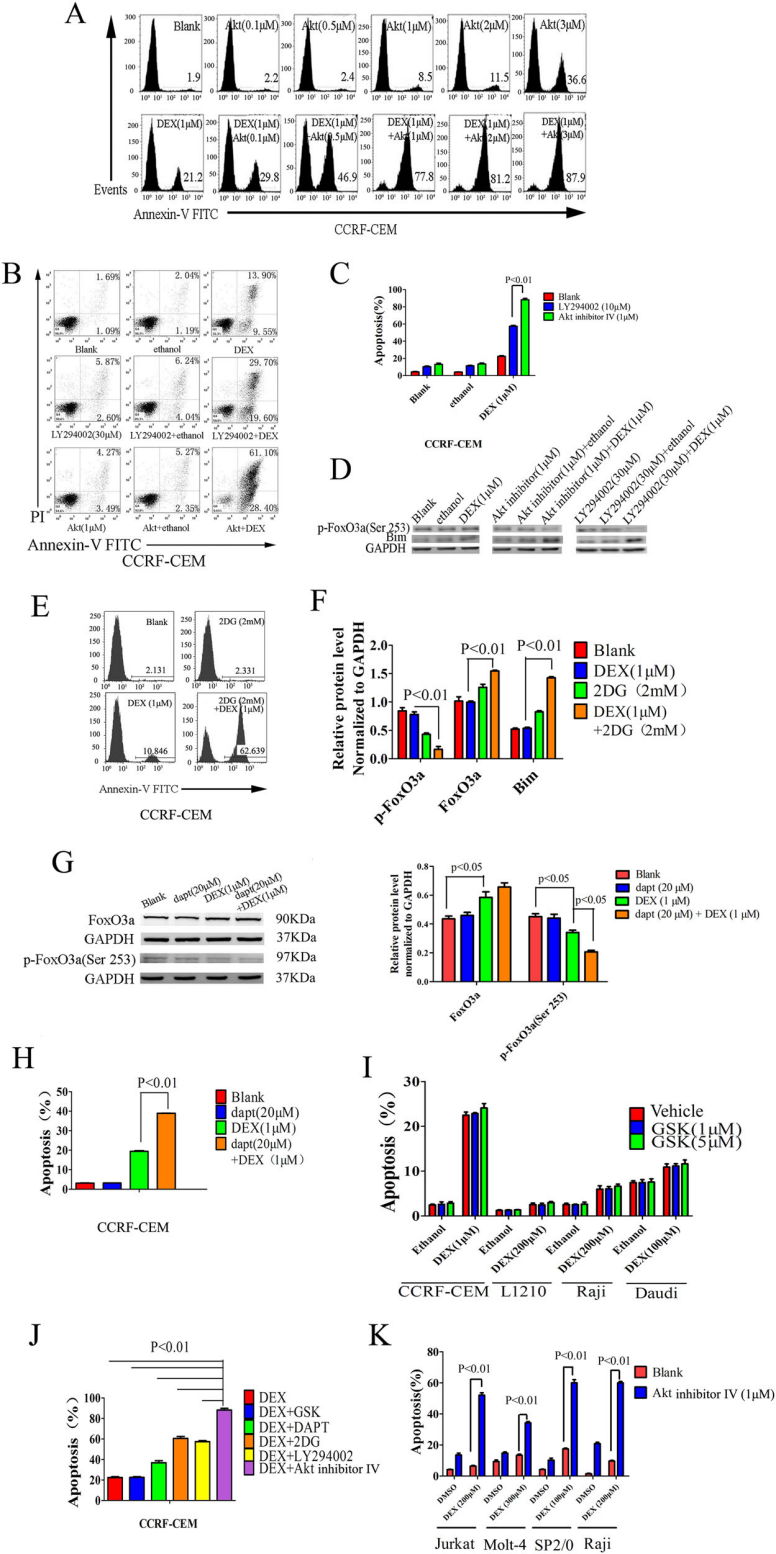
Inhibition of the Akt pathway enhances sensitivity to GCs. **a** Levels of apoptosis of cells treated with different concentrations of the Akt inhibitor or different concentrations of Akt inhibitor combined with DEX were measured using flow cytometry. **b**, **c** Apoptosis was measured by flow cytometry in CCRF-CEM cells treated with ethanol, DEX (1 µM), Akt inhibitor (1 µM), PI3K inhibitor LY294002 (30 µM), Akt inhibitor (1 µM) plus ethanol, LY294002 (30 µM) plus ethanol, Akt inhibitor (1 µM) plus DEX (1 µM) or LY294002 (30 µM) plus DEX (1 µM). **d** Levels of p-FoxO3a and Bim in CCRF-CEM cells treated with ethanol, DEX, Akt inhibitor, PI3K inhibitor, LY294002 or a combination of the inhibitors were examined by western blot with the indicated antibodies. GAPDH served as a loading control. **e** Apoptosis analysis using flow cytometry in CCRF-CEM T-ALL cells treated with the glycolysis inhibitor 2-DG (2 mM), DEX (1 µM) or 2-DG (2 mM) plus DEX (1 µM). **f** Relative levels of FoxO3a/Bim signaling proteins in CCRF-CEM cells treated with the glycolysis inhibitor 2-DG (2 mM), DEX (1 µM) or 2-DG (2 mM) plus DEX (1 µM) as measured by western blot. Levels were normalized to GAPDH. **g** Analysis of FoxO3a and p-FoxO3a (Ser253) levels in CCRF-CEM cells treated with the notch signaling inhibitor dapt (20 µM), DEX (1 µM) or dapt (20 µM) plus DEX (1 µM) by western blot. GAPDH served as a loading control. **h** Analysis of apoptosis using flow cytometry in CCRF-CEM T-ALL cells treated with the notch signaling inhibitor dapt (20 µM), DEX (1 µM) or dapt (20 µM) plus DEX (1 µM). **i** Analysis of apoptosis using flow cytometry in CCRF-CEM T-ALL cells, L1210 leukemia cells from mice, Raji or Daudi Burkitt’s lymphoma cells treated with ethanol plus vehicle, SGK inhibitor GSK (1, 5 µM) plus ethanol, vehicle plus DEX or GSK (1, 5 µM) plus DEX. **j** Analysis of apoptosis by flow cytometry in CCRF-CEM T-ALL cells treated with different pathway inhibitors including GSK, dapt, 2-DG, LY294002 and Akt IV combined with DEX (1 µM). **k** Apoptosis was measured by flow cytometry in Jurkat T-ALL cells, Molt-4 T-ALL cells, SP2/0 myeloma cells from mice or Raji Burkitt’s lymphoma cells treated with DMSO only, Akt inhibitor (1 µM), DEX or Akt inhibitor (1 µM) combined with DEX. Bar graphs in **c**, **f**, **g**–**k** represent mean ± SD

To examine the therapeutic role of Akt inhibitors in the treatment of GC-resistant leukemias, we treated CCRF-CEM cells with DEX and an Akt inhibitor, Akt inhibitor IV. Analysis using flow cytometry shows that treatment of CCRF-CEM lymphoblasts with Akt inhibitor IV effectively restores GC-induced apoptosis and reverses GC resistance in vitro and found that 1 µM Akt inhibitor IV with 1 µM DEX is the most effective combination (Fig. 2a). The PI3K inhibitor LY294002 can also increase sensitization of CCRF-CEM cells to DEX, but with a less significant effect than the Akt inhibitor (Fig. 2b, c). Treatment with these inhibitors results in decreased expression of p-FoxO3a and increased Bim expression (Fig. 2d). Additional pathways affect GC-induced apoptosis in lymphocytes. We selected the glycolysis inhibitor 2-DG to measure its effect on tumor cell apoptosis. Prednisolone resistance is associated with an increased glucose consumption, and the use of 2-DG sensitizes prednisolone-resistant ALL cell lines to GCs^21^. We found that 2-DG sensitizes CCRF-CEM cells to DEX and results in increased expression of FoxO3a and Bim and decreased expression of p-FoxO3a (Fig. 2e, f). It was previously reported that NOTCH1 activating mutations occur in >50% of cases of human T-ALL^23^. Notch signaling was shown to antagonize GC-induced apoptosis in T-lymphocytes^24^. In CCRF-CEM cells, the Notch signaling inhibitor dapt increases apoptosis and decreases p-FoxO3a expression in the presence of DEX (Fig. 2g, h). Serum/glucocorticoid-regulated kinases (SGKs) are activated by the phosphoinositide-3 kinase (PI3K) and translocate to the nucleus upon growth factor stimulation^23^. While there are no reports regarding the relationship between SGKs and GC resistance, Náray-Fejes-Tóth and colleagues found that SGK mRNA levels in lymphocytes are markedly induced by GCs^25^. We, therefore, tested the GC-sensitizing effects of the SGK inhibitor GSK in T and B lymphocytes. Our results show that the SGK inhibitor could not restore sensitivity to DEX either in resistant T-cell or B-cell tumors (Fig. 2i).

A comparison of the aforementioned pathway inhibitors with regards to their efficiency in restoring sensitivity to DEX in CCRF-CEM cells shows that the Akt inhibitor was most effective at inducing apoptosis in the presence of DEX (Fig. 2j). At a concentration of 1 µM, the Akt inhibitor combined with DEX restores sensitivity to DEX in the GC-resistant Molt-4 and Jurkat T-ALL cell lines as well as the resistant B-cell tumors SP2/0 and Raji cell lines (Fig. 2k).

### An inhibitor of Akt resensitizes tumors to GC in vivo but is associated with significant liver toxicity

**Fig. 3:**
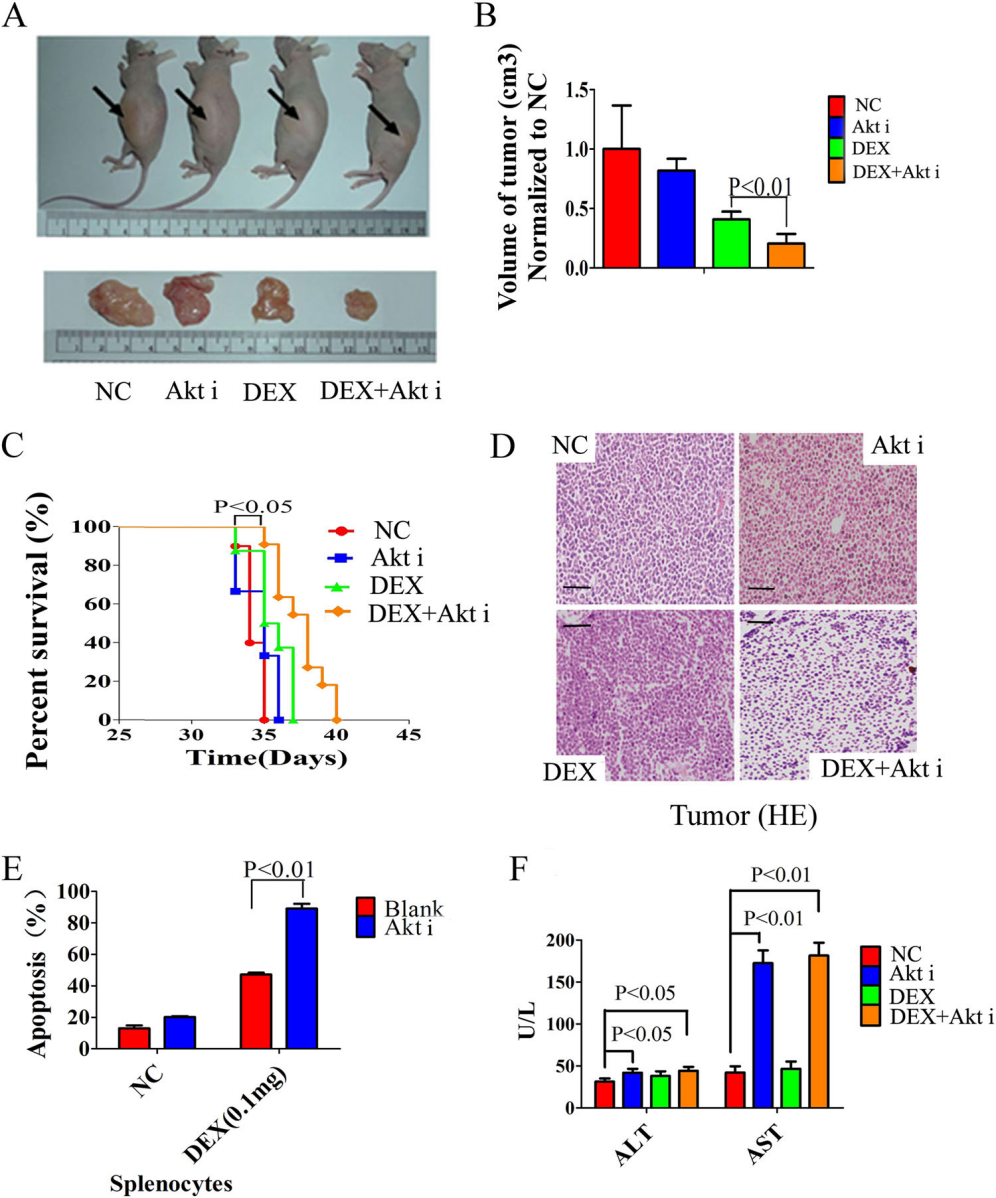
The Akt inhibitor resensitizes tumors to GC in vivo but is associated with significant liver toxicity. **a**, **b** Volumes of CCRF-CEM xenograft tumors in nude mice injected intraperitoneally with saline (negative control, NC), Akt inhibitor IV (1.25 µg), DEX (0.1 mg), or Akt inhibitor IV (1.25 µg) combined with DEX (0.1 mg) for 7 days. **c** Kaplan–Meier curve of tumor-bearing mice treated with saline (negative control, NC), Akt inhibitor IV (1.25 µg), DEX (0.1 mg) or Akt inhibitor IV (1.25 µg) combined with DEX (0.1 mg). **d** Pathological histology of CCRF-CEM xenograft tumors in nude mice injected intraperitoneally with saline (negative control, NC), Akt inhibitor IV (1.25 µg), DEX (0.1 mg) or Akt inhibitor IV (1.25 µg) combined with DEX (0.1 mg) for 7 days. Scale bar: 100 µm. **e** Analysis of apoptosis of splenocytes in tumor-bearing mice treated with saline (negative control, NC), Akt inhibitor IV (1.25 µg), DEX (0.1 mg) or Akt inhibitor IV (1.25 µg) combined with DEX (0.1 mg). **f** Detection of liver enzyme ALT and AST in peripheral blood of tumor-bearing mice treated with saline (negative control, NC), Akt inhibitor IV (1.25 µg), DEX (0.1 mg), or Akt inhibitor IV (1.25 µg) combined with DEX (0.1 mg). Bar graphs in **b**, **e**, **f** represent mean ± SD

We implanted CCRF-CEM cells subcutaneously into nude mice to induce tumor formation. Animals with similar tumor volumes were then injected intraperitoneally with saline, Akt inhibitor IV, DEX or Akt inhibitor IV combined with DEX for 7 days. We found that Akt inhibitor IV combined with DEX could significantly minimize the tumors (Fig. 3a, b) and prolong the survival time of the mice (Fig. 3c). We also found this therapy significantly increased GC-induced apoptosis of tumor cells and splenocytes in tumor-bearing mice (Fig. 3d, e), while Akt inhibitor IV or DEX alone did not achieve these results. Akt signaling pathways are widely distributed in cells, playing an important role in the growth, differentiation, and proliferation of cells^26,27^. To further explore effects of inhibition of Akt on liver cells, we examined the liver enzymes ALT and AST in peripheral blood of mice treated with the Akt inhibitor. Compared with the saline treated group, Akt inhibitor-treated mice show severe liver damage with ALT and AST levels increasing significantly (Fig. 3f).

### Overexpression of Akt2, as a major negative regulator of the FoxO3a activity, mediates GC resistance in lymphocytes

**Fig. 4:**
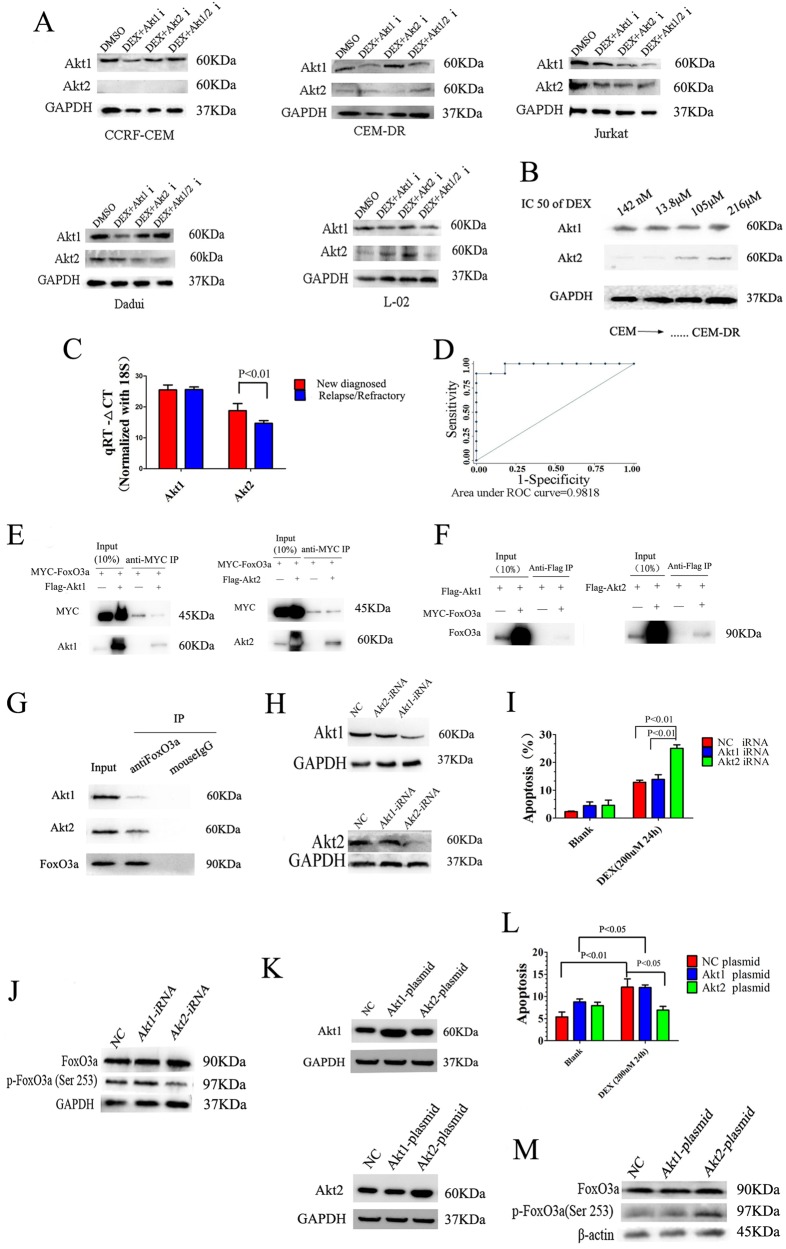
Overexpression of Akt2, as a major negative regulator of the FoxO3a activity, mediates GC resistance in lymphocytes. **a** Western blot analysis of Akt1 and Akt2 expression in CCRF-CEM cells, CEM-DR cells, Jurakt cells, Daudi cells, and L-02 cells. **b** Western blot analysis of Akt1 and Akt2 expression in CCRF-CEM cells with increasing of IC50 value of DEX. GAPDH was used as a loading control. **c** Real-time quantitative PCR analysis of Akt1 and Akt2 mRNA in lymphoctyes from bone marrow of newly diagnosed or relapse/refractory ALL patients. **d** Diagnostic sensitivity and specificity analysis of Akt2 mRNA as an indicator of GC resistance in ALL patients. **e** Western blot analysis of Akt1/Akt2 and MYC after FoxO3a immunoprecipitation in 293T cells expressing Flag-tagged Akt1/Akt2 and MYC-tagged FoxO3a. **f** FoxO3a western blot analysis after Akt1/Akt2 immunoprecipitation in 293T cells expressing Flag-tagged Akt1/Akt2 and MYC-tagged NR3C1. **g** Western blot analysis of Akt1 or Akt2 after FoxO3a protein immunoprecipitation in Jurkat cells. **h** Western blot analysis of Akt1 and Akt2 expression in Jurkat T-ALL cells after Akt1 iRNA or Akt2 iRNA transfection. **i** Analysis of apoptosis in Jurkat T-ALL cells after Akt1 iRNA or Akt2 iRNA transfection with 24 h incubation with DEX. **j** Western blot analysis of FoxO3a and p-FoxO3a (Ser253) expressions in Jurkat T-ALL cells after Akt1 iRNA or Akt2 iRNA transfection with 24 h incubation with DEX. **k** Western blot analysis of Akt1 and Akt2 expression in Jurkat T-ALL cells after Akt1 plasmid or Akt2 plasmid transfection. **l** Analysis of apoptosis in Jurkat T-ALL cells after Akt1 plasmid or Akt2 plasmid transfection with 24 h incubation with DEX. **m** Western blot analysis of FoxO3a and p-FoxO3a (Ser253) expressions in Jurkat T-ALL cells after Akt1 plasmid or Akt2 plasmid transfection with 24 h incubation with DEX. Bar graphs in **c**, **i**, **l** represent mean ± SD

Our preliminary results have shown that CCRF-CEM cells are relatively sensitive to GCs; CEM-DR cells derived from CCRF-CEM cells are highly resistant to GCs after DEX stimulation; Jurkat and Daudi are intrinsically highly resistant to GCs. In addition to these cells, we selected a human liver cell line, L-02, which is considered as a natural GC-resistant cell line without the effect of GC-induced apoptosis. To explore whether Akt1 or Akt2 expression is related to GC resistance, we examined Akt1 and Akt2 expression in the aforementioned cell lines. Western blot analysis shows that Akt1 expression in all five cell lines is similar. Akt2 expression in CEM-DR, Jurkat, Daudi, and L-02 cells is significantly higher than in CCRF-CEM cells (Fig. 4a). Then we used the CCK-8 assay to determine the degree of resistance to DEX of CCRF-CEM cells at different stages of resistance construction process. Along with the half maximal inhibitory concentration (IC50) of DEX in CCRF-CEM cells increases, the expression of Akt2 increases while the expression of Akt1 did not change (Fig. 4b). To further explore if the overexpression of Akt2 is correlated with GC resistance in lymphocytes in a clinical context, we tested Akt1 and Akt2 mRNA levels in lymphoctyes from the bone marrow of 21 ALL patients, including 10 newly diagnosed patients and 11 relapsed or refractory patients (after an average of 7.2 courses of GC-containing treatment). Compared with the newly diagnosed group, the relapsed/refractory group has higher Akt2 mRNA expression while Akt1 mRNA expression did not differ significantly between the two groups (Fig. 4c). We used the receiver operating characteristic (ROC) curve to analyze if Akt2 mRNA could be an indicator of GC resistance in a clinical setting. The area under the ROC curve was 0.9818 and the optimal threshold is 16.39 with a diagnostic sensitivity of 90% and specificity of 100% (Fig. 4d).

To detect if Akt2 was able to directly interact with FoxO3a, co-immunoprecipitation assay was employed. MYC-FoxO3a was co-transfected with Flag-Akt1 or Flag-Akt2 into HEK293T cells. The reaction product was first immunoprecipitated with anti-MYC antibody and then blotted with anti-MYC and anti-Akt1 or Akt2 antibodies. Western blot analysis demonstrated the more presence of Flag-AKT2 than Flag-Akt1 in MYC-FoxO3a immunoprecipitates, suggesting that AKT2 as the major regulator directly interacting with FoxO3a (Fig. 4e). Reciprocal immunoprecipitation experiments confirmed the closer association between Flag-Akt2 and MYC-FoxO3a (Fig. 4f). Moreover, immunoprecipitation of FoxO3a protein complexes from the Jurkat T-ALL cell line demonstrated that endogenous FoxO3a can interact closer with Akt2 than with Akt1 in T-ALL lymphoblast cells (Fig. 4g).

Next, we used siRNA to downregulate Akt1 or Akt2 expression in GC-resistant Jurkat cells (Fig. 4h). We found that GC-induced apoptosis increased significantly after downregulation of Akt2 expression, along with the expression of p-FoxO3a (Ser253) decreased. Furthermore, the level of GC-induced apoptosis and the expression of p-FoxO3a (Ser253) did not change after downregulation of Akt1 expression (Fig. 4i, j). Then we transfected Jurkat cells with plasmid constructs increasing the expression of Akt1 and Akt2 (Fig. 4k), and also found that GC-induced apoptosis significantly decreased and the expression of p-FoxO3a (Ser253) increased after upregulation of Akt2 expression, while GC-induced apoptosis and the expression of p-FoxO3a did not change after upregulation of Akt1 expression (Fig. 4l, m).

### Inhibition of Akt2 more significantly enhances sensitivity to GCs than inhibition of Akt1 in T-cell tumors in vitro

**Fig. 5:**
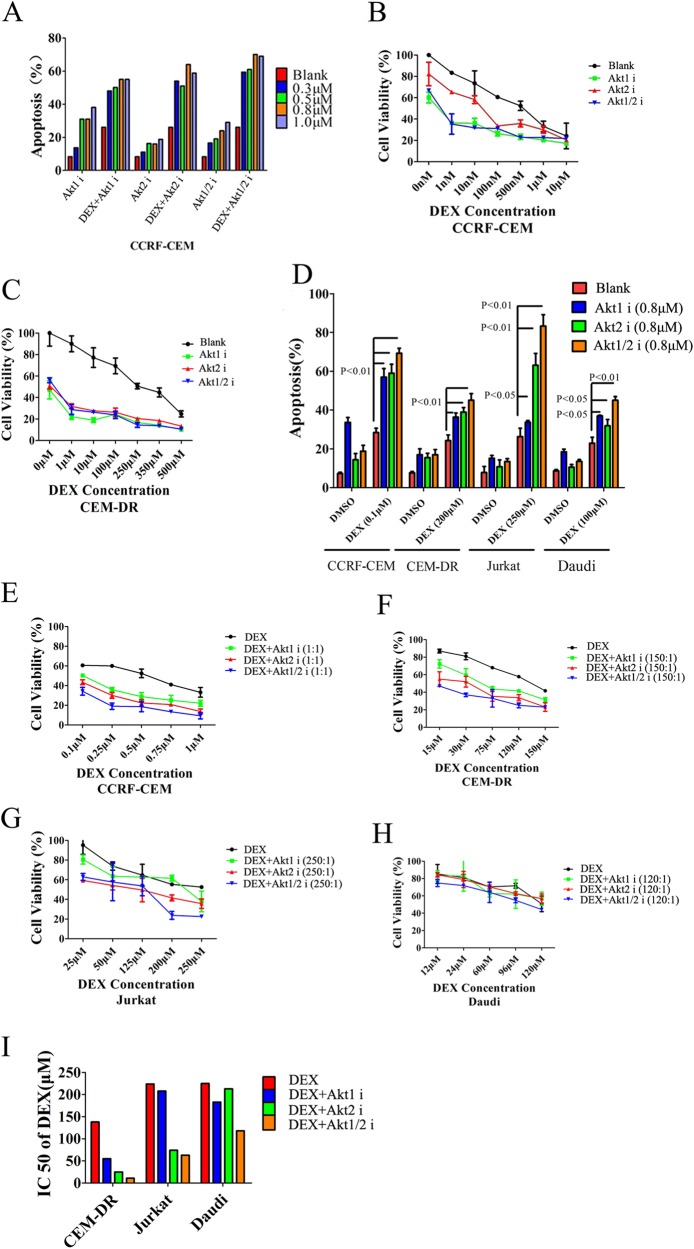
Inhibition of Akt2 enhances sensitivity to GCs in T-cell tumors to a greater extent than inhibition of Akt1. **a** Level of apoptosis in CCRF-CEM cells when cells were treated with DEX (0.1 µM), varying concentrations of the Akt1, Akt2, or Akt1/2 inhibitor (0.3, 0.5, 0.8, or 1 µM), or DEX combined with varying concentration of each of the Akt isoform inhibitors. **b** Quantification of cell viability of CCRF-CEM T-ALL cells treated with varying concentrations of DEX plus each of the Akt isoform inhibitors (0.8 µM). **c** Cell viability quantification in CEM-DR cells treated with different concentrations of DEX at indicated concentrations plus each of the Akt isoform inhibitors at a concentration of 0.8 µM. **d** Analysis of apoptosis in CCRF-CEM T-ALL cells, CEM-DR T-ALL cells, Jurkat T-ALL cells and Daudi Burkitt’s lymphoma cells treated with DEX, each of the Akt isoform inhibitors at 0.8 µM, or DEX combined with each of the Akt isoform inhibitors. **e**–**h** Cell viability quantification in CCRF-CEM cells, CEM-DR cells, Jurkat cells and Daudi cells treated with a fixed concentration ratio of DEX and each Akt isoform inhibitor. **i** IC50 of DEX in CEM-DR cells, Jurkat cells, and Daudi cells treated with DEX alone or DEX combined with the Akt1 inhibitor, Akt2 inhibitor or Akt1/2 inhibitor. Data in **b**, **c**, **e**–**h** show average measurements of triplicate biological replicas and error bars indicate ± SD. Bar graph in **d** represents mean ± SD

To examine the therapeutic role of Akt isoform specific inhibitors in the treatment of GC-resistant lymphoid tumor cells, we treated CCRF-CEM cells with DEX and A-674563 (Akt1 inhibitor), CCT128930 (Akt2 inhibitor), or Akti1/2 (Akt1/2 inhibitor). When exploring the appropriate dose of Akt isoform inhibitors with DEX in CCRF-CEM cells, we found that a concentration of 0.8 µM is the most effective dose of Akt isoform inhibitors in the presence of 0.1 µM DEX (Fig. 5a). In combination with varying concentrations of DEX from 0 to 500 nM, the Akt isoform inhibitors significantly decrease the viability of CCRF-CEM cells (Fig. 5b). Similar result was obtained in the highly GC-resistant CEM-DR cell line (Fig. 5c). Flow cytometry experiments demonstrate inhibition of Akt1 or Akt2 in CCRF-CEM, CEM-DR, Jurkat, and highly GC-resistant Burkitt’s lymphoma cell line Daudi with a concentration of 0.8 µM Akt isoform inhibitors effectively restores GC-induced apoptosis. Remarkably, in Jurkat cells, Akt2 inhibitor or Akt1/2 inhibitor shows higher GC-induced apoptosis than Akt1 inhibitor (Fig. 5d). To further test the GC-sensitizing effects of Akt isoform inhibitors, we used the CCK-8 assay to test the viability of aforementioned cell lines following treatment with a fixed concentration ratio of DEX and each Akt isoform inhibitor (Fig. 5e–h). The IC50 values of DEX in aforementioned cell lines demonstrate that Akt isoform inhibitors could effectively restore sensitive to GC, especially in T-cell tumor cell lines and Akt2 inhibitor has more effective GC-sensitizing effects than Akt1 inhibitor with a lower IC50 (Fig. 5i and Table 1). Next, we used the CompuSyn method^28^ to calculate the combination index (CI) to further determine the GC-sensitizing effects of the combinations (Table 1). Our analysis shows that each of the Akt isoform inhibitors act synergistically with DEX to increase apoptosis in the T-cell tumor cells. Synergy of DEX with the Akt2 inhibitor is superior to that of DEX and the Akt1 inhibitor, especially in the Jurkat cell line. While in GC-resistant B-cell line Daudi, there is no apparent synergy between DEX and the Akt isoform inhibitors.

**Table 1:** The IC50 of DEX (μM) and combination index (CI) in lymphoid tumor cell lines when treated with an appropriate concentration ratio of combined with Akt1 inhibitor, Akt2 inhibitor, or Akt1/2 inhibitor, respectively

Lymphoid tumor cell line	The concentration ratio (DEX: Akt isoform inhibitors)	IC50 (DEX)	DEX+Akt1			DEX+Akt2			DEX+Akt1/2		
			IC50	CI	Synergistic interaction	IC50	CI	Synergistic interaction	IC50	CI	Synergistic interaction
CCRF-CEM	1:1	0.3	0.18	0.59	+	0.13	0.27	+ + +	0.03	0.15	+ + + +
CEM-DR	150:1	138	55	0.87	+ -	25	0.25	+ + +	11	0.08	+ + + +
Jurkat	250:1	224	208	1.2	-	74	0.38	+ + +	63	0.39	+ + +
Daudi	120:1	225	183	0.88	+ -	213	0.97	-	118	0.73	+

A combination index (CI) below 1.0 indicates a synergistic interaction, equal to 1.0 indicates an additive interaction and greater than 1 indicates an antagonistic interaction^28^

### Inhibition of Akt2 resensitizes GC-resistant cells by enhancing FoxO3a/Bim signaling pathways in lymphocyte

**Fig. 6:**
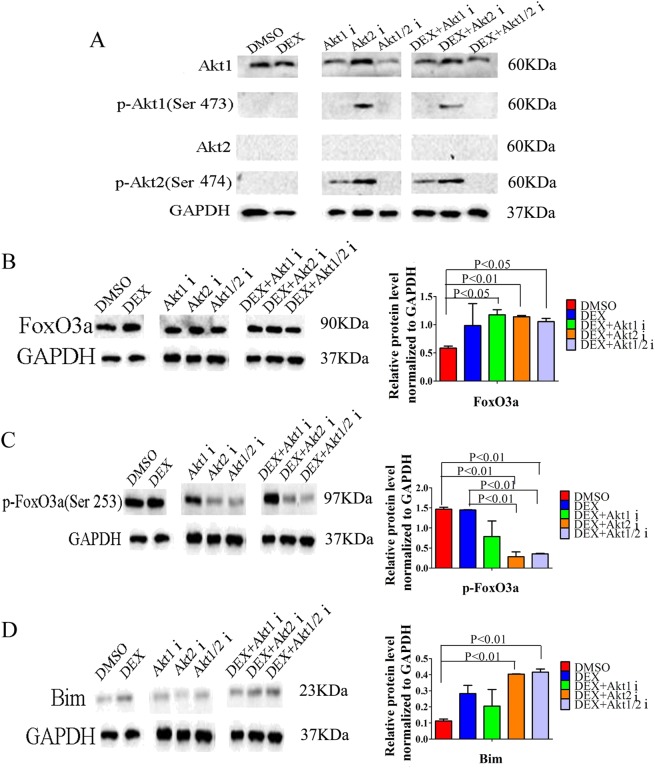
The Akt2 inhibitor enhances the FoxO3a/Bim signaling pathway in lymphocytes and reverses GC resistance. **a** Analysis by western blot of Akt1, p-Akt1 (Ser473), Akt2, and p-Akt2 (Ser474) in CCRF-CEM ALL cells treated with DEX, the Akt isoform inhibitors, or DEX plus the Akt inhibitors. **b**–**d** Western blot analysis and relative quantification of FoxO3a/Bim signaling proteins in CCRF-CEM ALL cells treated with DEX, Akt1 inhibitor, Akt2 inhibitor or Akt1/2 inhibitor, or DEX plus Akt1 inhibitor, Akt2 inhibitor or Akt1/2 inhibitor. Bar graphs in **b**–**d** represent mean ± SD

Increases in p-Akt1 expression are observed by western blot analysis following incubation with the Akt2 inhibitor, while p-Akt2 expression increases after incubation with the Akt1 or Akt2 inhibitor (Fig. 6a). When we tested the expression of FoxO3a/Bim signaling proteins in CCRF-CEM cells, there were no differences between the DEX treatment group and the Akt isoform inhibitors plus DEX groups in terms of expression of FoxO3a (Fig. 6b). The Akt2 or Akt1/2 inhibitor combined with DEX could significantly decrease the expression of p-FoxO3a (Ser253) and increase the expression of the pro-apoptotic protein Bim (Fig. 6c, d). The Akt1 inhibitor combined with DEX did not alter the expression levels of p-FoxO3a (Ser253) and Bim when compared to DEX alone (Fig. 6c, d).

### Inhibition of Akt2 reverses GC resistance in vivo

**Fig. 7:**
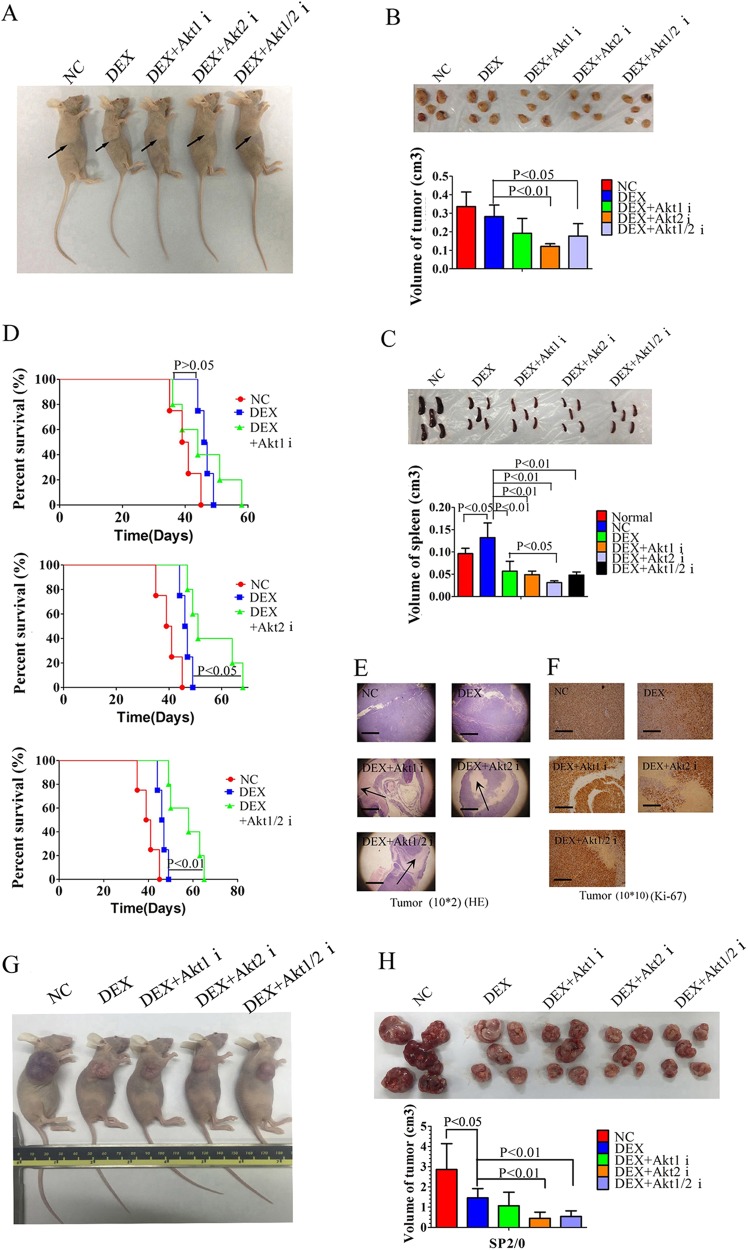

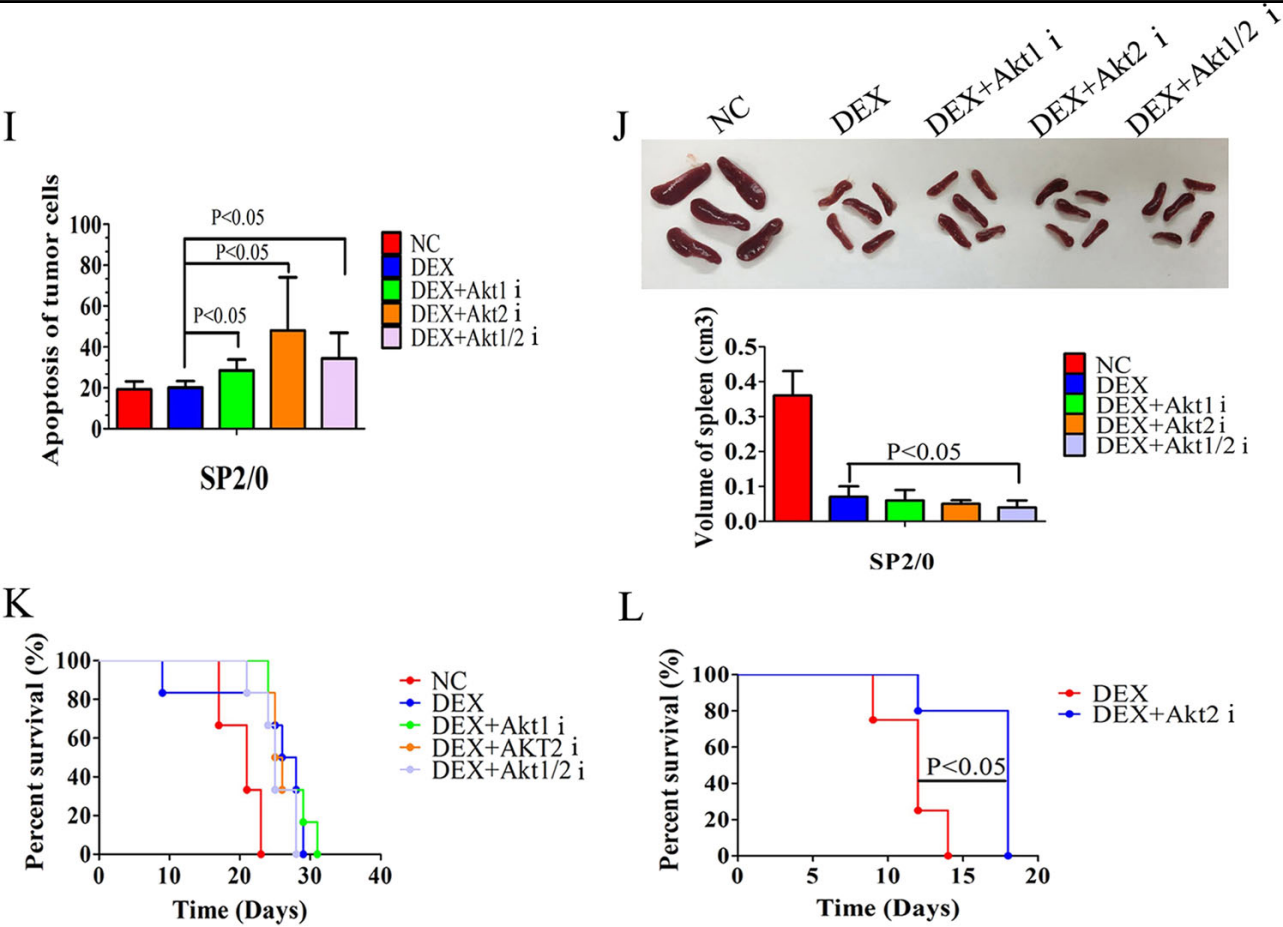
Inhibition of Akt2 effectively reverses GC resistance in vivo. **a**, **b** Volumes of CCRF-CEM xenograft tumors in nude mice injected intraperitoneally with saline (negative control, NC), DEX (0.1 mg), DEX plus each of the Akt isoform inhibitors (2 × 10^−3^ µmol) for 7 days. **c** Volumes of spleens in nude mice or tumor-bearing mice injected intraperitoneally with saline (negative control, NC), DEX (0.1 mg), DEX plus each of the Akt isoform inhibitors (2 × 10^−3^ µmol) for 7 days. **d** Kaplan–Meier curve of tumor-bearing mice treated with saline (negative control, NC), DEX (0.1 mg), DEX plus each of the Akt isoform inhibitors (2 × 10^−3^ µmol) **e**, **f** Pathological histology of CCRF-CEM xenograft tumors with HE staining or Ki-67 staining in tumor-bearing mice treated with saline (negative control, NC), DEX (0.1 mg), DEX plus each of the Akt isoform inhibitors (2 × 10^−3^ µmol), Scale bar: 2500 µm for **e**; 500 µm for **f**. **g**, **h** Volumes of SP2/0 allograft tumors in nude mice injected intraperitoneally with saline (negative control, NC), DEX (0.1 mg), DEX plus Akt1 inhibitor (2 × 10^−3^ µmol), DEX plus Akt2 inhibitor (2 × 10^−3^ µmol) or DEX plus Akt1/2 inhibitor (2 × 10^−3^ µmol) for 7 days. **i** Analysis of apoptosis in tumor cells of tumor-bearing mice injected intraperitoneally with saline (negative control, NC), DEX (0.1 mg), DEX plus Akt1 inhibitor (2 × 10^−3^ µmol), DEX plus Akt2 inhibitor (2 × 10^−3^ µmol) or DEX plus Akt1/2 inhibitor (2 × 10^−3^ µmol) for 7 days. **j** Volumes of spleens in tumor-bearing mice injected intraperitoneally with saline (negative control, NC), DEX (0.1 mg), DEX plus Akt1 inhibitor (2 × 10^−3^ µmol), DEX plus Akt2 inhibitor (2 × 10^−3^ µmol) or DEX plus Akt1/2 inhibitor (2 × 10^−3^ µmol) for 7 days. **k** Kaplan–Meier curve of tumor-bearing mice treated with saline (negative control, NC), DEX (0.1 mg), DEX plus Akt1 inhibitor (2 × 10^−3^ µmol), DEX plus Akt2 inhibitor (2 × 10^−3^ µmol) or DEX plus Akt1/2 inhibitor (2 × 10^−3^ µmol) for 7 days. **l** Kaplan–Meier curve of tumor-bearing mice treated with DEX (0.1 mg) or DEX plus Akt1/2 inhibitor (2 × 10^−3^ µmol) for 11 days. Bar graphs in **h**–**j** represent mean ± SD

Using a subcutaneous xenograft model of CCRF-CEM cells in nude mice, we analyzed the antitumor effects of Akt isoform inhibitors combined with DEX. Animals harboring homogeneous tumor burdens were treated with normal saline (negative control, NC), DEX or Akt isoform inhibitors plus DEX for 7 days. In this experiment, treatment with the Akt2 or Akt1/2 inhibitor plus DEX shows a significant anti-leukemic effect with reduced tumor size and prolonged overall survival of mice when compared with DEX alone, while treatment with the Akt1 inhibitor plus DEX did not achieve these results (Fig. 7a, b, d). Only the combination of the Akt2 inhibitor plus DEX shows decreased spleen size when compared to the DEX alone group (Fig. 7c). The tumors highly expressing Ki-67 show large areas of necrosis when treated with DEX plus each of the Akt isoform inhibitors (Fig. 7e, f). In spleens of tumor-bearing mice, treatment with DEX alone significantly reduces human leukemia cells highly expressing CD3 and TdT. The effect is slightly weaker than the combination of DEX with each of Akt isoform inhibitors (Supplementary Fig. S1B, C).

To further test the therapeutic role of Akt isoform inhibition in GC-resistant B lymphocytes in vivo, we established an allograft model of SP2/0 cell line, a GC-resistant myeloma cell line of mouse. In this experiment, animals treated with DEX combined with Akt2 or Akt1/2 inhibitor show significantly reduced tumor size similar to those observed in CCRF-CEM xenograft model (Fig. 7g, h). Akt isoform inhibitors significantly increase the GC-induced apoptosis of tumor cells in mice (Fig. 7i). Only treatment with Akt1/2 inhibitor plus DEX decreases spleen size when compared to the DEX alone group (Fig. 7j). Seven days of treatment of Akt isoform inhibitors plus DEX could not prolong the overall survival of tumor-bearing mice (Fig. 7k). When the time of treatment extended to 11 days, Akt2 inhibitor plus DEX prolong the overall survival of mice (Fig. 7l).

### Inhibition of Akt2 shows less liver toxicity than inhibition of Akt1

**Fig. 8:**
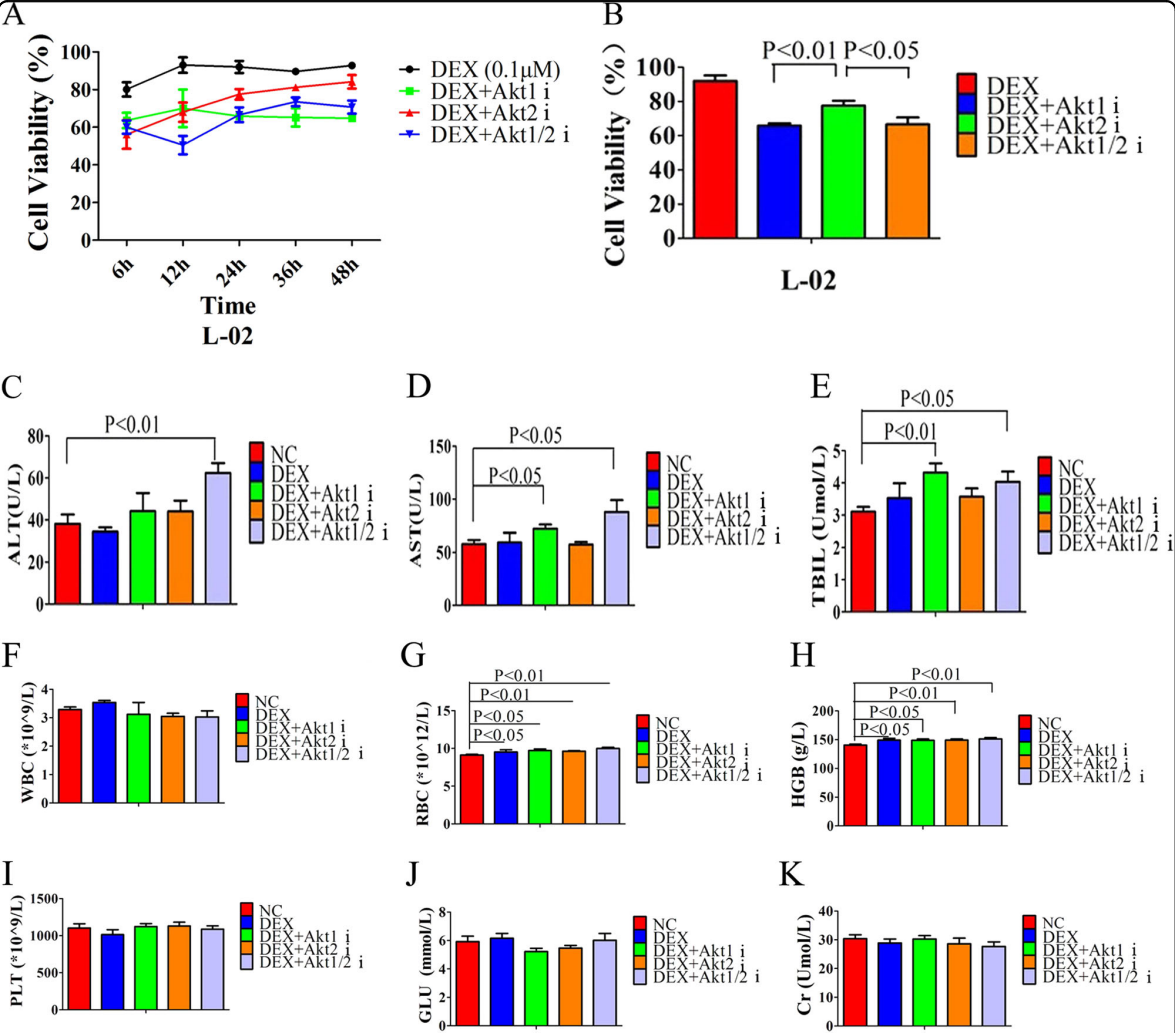
Inhibition of Akt2 shows less liver toxicity than inhibition of Akt1. **a** Quantification of cell viability in L-02 cells after 0, 12, 24, 36, and 48 h incubation with DEX, DEX plus Akt1 inhibitor, DEX plus Akt2 inhibitor or DEX plus Akt1/2 inhibitor. **b** Quantification of cell viability in L-02 cells after 24 h incubation with DEX, DEX plus Akt1 inhibitor, DEX plus Akt2 inhibitor or DEX plus Akt1/2 inhibitor. **c**–**e** Liver function indices. ALT, AST, or TBIL from peripheral blood of nude mice injected intraperitoneally with saline (negative control, NC), DEX (0.1 mg), DEX plus each of the Akt isoform inhibitors (2 × 10^−3^ µmol) for 7 days. **f**–**i** Routine blood indices white blood cells, red blood cells, blood red lead, and platelets from peripheral blood of nude mice injected intraperitoneally by saline (negative control, NC), DEX (0.1 mg), DEX plus Akt1 inhibitor (1.25 µg), DEX plus Akt2 inhibitor (1.25 µg) or DEX plus Akt1/2 inhibitor (1.25 µg) for 7 days. **j** Blood glucose levels of peripheral blood in nude mice injected intraperitoneally by saline (negative control, NC), DEX (0.1 mg), DEX plus Akt1 inhibitor (1.25 µg), DEX plus Akt2 inhibitor (1.25 µg), or DEX plus Akt1/2 inhibitor (1.25 µg) for 7 days. **k** Creatinine levels in peripheral blood from nude mice injected intraperitoneally by saline (negative control, NC), DEX (0.1 mg), DEX plus Akt1 inhibitor (1.25 µg), DEX plus Akt2 inhibitor (1.25 µg), or DEX plus Akt1/2 inhibitor (1.25 µg) for 7 days. Bar graphs in **b**–**k** represent mean ± SD

When exploring the influence on liver cell’s viability of Akt1, Akt2, or Akt1/2 inhibitors in vitro, we measured L-02 cell viability following a 6, 12, 24, 36, or 48 h incubation with each inhibitor. Our data shows the influence on liver cell’s viability by Akt2 inhibitor is minimal: cell viability in this group is gradually restored and by 24 h always higher than that in Akt1 and Akt1/2 inhibitor groups (Fig. 8a, b). To further test the damage to liver by Akt isoform inhibitors in vivo, we found that the Akt1 inhibitor leads to higher AST and total bilirubin (TBIL) levels, the Akt1/2 inhibitor leads to increased ALT, AST, and TBIL levels, while the Akt2 inhibitor did not show significantly increased liver function indices when compared with negative control group, indicating that inhibition of Akt2 has milder liver toxicity in vivo (Fig. 8c–e). Western blot analysis demonstrate that the Akt isoform inhibitors also enhance the FoxO3a/Bim signaling pathway in liver cell by increasing FoxO3a expression, lowering p-Foxo3a expression and increasing Bim expression (Supplementary Fig. S2).

When exploring the influence on other viscera functions by Akt isoform inhibitors in vivo, we found that Akt isoform inhibitors did not change white blood cell (WBC) count, platelet (PLT) count, blood glucose or creatinine (Cr) level of nude mice (Supplementary Fig. 8F, I–K). Due to the DEX stimulation, red blood cells (RBCs) from the reserve pool transfer to the circular pool then RBC count and hemoglobin (HGB) levels increase (Supplementary Fig. 8G, H). We also examined the pathological changes of Akt isoform inhibitors in vital organs and found that Akt isoform inhibitors did not cause the morphological changes in heart, kidneys, liver, or lungs of mice (Supplementary Fig. S3).

## Discussion

GCs are common components in many chemotherapeutic protocols for lymphoid malignancies. Although they are effective in the initial stages, patients often develop resistance to GCs on relapse. Recently, some studies have proposed that the molecular mechanisms of resistance to GCs in ALL may be associated with RAS-pathway activating mutations^29,30^ and NALP3 inflammasome upregulation and CASP1 cleavage of the GC receptor^31^. Li et al.^32^ performed genome sequencing on pre-treatment and post-treatment samples from 82 pediatric T-ALL patients, and found that mutations in IL-7R signaling pathway genes including JAK1, KRAS, IL-7R, JAK3, NF1, NRAS, and Akt were associated with both GC resistance and poor outcomes. Therapeutic targeting of the IL-7Ra signaling pathways including the Akt inhibitors were also recommended in ALL treatment^33^, but the cellular mechanisms of Akt signaling pathway underlying GC resistance were not fully elucidated in this study.

In our current study, we chose inhibitors of the PI3K pathway, Akt pathway, glycolysis pathway, NOTCH1 pathway and SGKs pathway, which have been reported to affect GC-induced apoptosis in lymphocytes^21,23–25^ to test their effects on GC-sensitization. We found all these pathway inhibitors enhance the FoxO3a/Bim pathway to reverse GC resistance, which further confirms that the FoxO3a/Bim pathway is essential for the development of GC resistance in lymphocytes. Through the analysis of the role of Akt isoforms Akt1 and Akt2 in the GC resistance mechanism, we found Akt2 is the major kinase phosphorylating FoxO3a and the expression of Akt2 in vitro or vivo correlates negatively with sensitive to GCs. Selective inhibition of Akt2 enhances the FoxO3a/Bim signaling pathway and more significantly restores the sensitivity to GCs than selective inhibition of Akt1. According to our result that Akt2 expression increases in GC-resistant lymphocytes especially secondarily GC-resistant CEM-DR cells, we further concluded that Akt2/FoxO3a/Bim signaling pathway is the mechanism of the gradual acquisition of GC resistance in lymphocytes repeatedly treated with hormones, and Akt2 not Akt1 may be the direct reversal target of GC resistance in lymphoid malignancy.

Inhibition of Akt with the Akt inhibitor IV in our study was shown to have a remarkable liver toxicity and other Akt inhibitors^10–12^ such as perifosine^10^ are also reported to be associated with the common hematologic and hepatic toxicities in clinical, negatively affecting their prospects for clinical applications. When testing the toxicity of Akt isoform inhibitors, we found that the Akt2 inhibitor has milder liver toxicity than the Akt1 or Akt1/2 inhibitors. This may be explained by the obvious differences in enzyme function of Akt1 and Akt2: Akt1 is ubiquitously expressed and plays an important role in cell proliferation^16,17^ while Akt2 is expressed at high levels in skeletal muscle and brown fat and is involved in the regulation of blood sugar^16–18^. Inhibition of Akt1 results in suppression of downstream proliferation pathways such as the mTOR pathway, especially in some eugonic cells such as liver cells and bone marrow cells; while inhibition of Akt2 may have more influence on the glucose metabolism and may be correlated with the reported hyperglycemia of Akt inhibitors in clinical^10–12^.

As inhibition of Akt2 can effectively reverse GC resistance, they may represent a novel and promising treatment to overcome GC resistance in lymphoid tumors. An Akt2 inhibitor combined with GCs may be used in the future for treatment of GC-resistant lymphoid tumors.

## Materials and methods

### Inhibitors and drugs

The phosphoinositide-3 kinase (PI3K) inhibitor LY294002, the glycolysis inhibitor 2-deoxy-d-glucose (2-DG), the Notch signaling inhibitor dapt and the serum/glucocorticoid-regulated kinases (SGKs) inhibitor GSK have been reported to increase GC-induced apoptosis of lymphocytes by inhibition of relevant signaling pathways. 2-DG and dapt were obtained from Sigma Corp. Akt inhibitor IV, GSK and LY294002 were purchased from Calbiochem, Tocris Bioscience and Promega Corp, respectively. Akt1 inhibitor A-674563 and Akt2 inhibitor CCT128930 were purchased from Selleckchem Corp. Akt1/2 inhibitor Akti 1/2 was obtained from Santa Cruz Corp. DEX was purchased from Sigma–Aldrich Corp.

### Cell lines

The mouse lymphocytic leukemia cell line L1210, the human acute T-lymphoblastic leukemia cell line CCRF-CEM, the human acute T-cell leukemia cell line Molt-4, the human acute T-cell leukemia cell line Jurkat, the human Burkitt’s lymphoma cell line Daudi and Raji, the mouse myeloma cell line SP2/0 and the human liver cell line L-02 were gifts from the cell bank of the Institute of Biochemistry and Cell Biology at the Chinese Academy of Sciences, Shanghai.

### Patient samples

Twenty-one ALL samples from 10 newly diagnosed patients and 11 relapsed/refractory patients (after an average of 7.2 courses of GC-containing treatment) were selected. Approval for the study was obtained from the Human Research Committee of Huadong Hospital Affiliated to Fudan University, and informed consent was obtained in accordance with the Declaration of Helsinki.

### Co-immunoprecipitation assay

HEK293T cells grown at 80% confluency were transfected with Turbofect. Cells were co-transfected with plasmid vectors containing MYC-FoxO3a and Flag-Akt1/Flag-Akt2. Single transfection with one of the plasmids alone was served as control. Twenty-four hours later, cells were lysed on ice by using 500 μl RIPA lysis buffer. The detergent soluble fraction was recovered by centrifugation at 4 °C for 20 min at 12,000 r/min and supernatants were subjected to immunoprecipitation with mouse anti-MYC or anti-Flag antibody. Immune complexes were isolated with protein G PLUS-Agarose beads. The immunoprecipitated products were washed four times with lysis buffer, eluted with 2 × sodium dodecyl sulfate–polyacrylamide gel electrophoresis loading buffer and analyzed by western blotting.

### Short-interfering RNA transfection

Short-interfering RNA (siRNA) targeting Akt1 and Akt2 were prepared by GenePharma corp. The sequences of siRNA targeting Akt1 were 5ʹ-GGCCCAACACCUUCAUCAUTT-3ʹ(sense) and 5ʹ -AUGAUGAAGGUGUUGGGCCTT-3ʹ(anti-sense). The sequences of siRNA targeting Akt2 were 5ʹ-GGUUCUUCCUCAGCAUCAATT-3ʹ(sense) and 5ʹ- UUGAUGCUGAGGAAGAACCTT-3ʹ(anti-sense). Scrambled siRNA was used as a control. Transfection of siRNAs was done according to the manufacturer’s protocol. The transfection efficiency of siRNA in Jurkat cells was more than 50% by flow cytometry detection.

### EGFP-tagged plasmid constructs of retroviral vector transfection

Enhanced green fluorescent protein (EGFP)-tagged plasmid constructs targeting Akt1 and Akt2 were prepared by GeneCopoeia corp. The plasmid of retroviral vector p-LXSN-Aktl, which contained the whole Aktl gene sequence, and p-LXSN-Akt2, which contained the whole Akt2 gene sequence, were used to transfect Jurkat cells with fuGENE according to the manufacturer’s protocol. The empty vector p-LXSN was used as a control. And then the positive cell clones were selected with antibiotics G418. The transfection efficiency of retroviral vector in Jurkat cells was 30–40% by flow cytometry detection.

### Establishment of tumors in mice

The animal protocol was approved by the institutional animal use committee of the Shanghai Institutes for Biological Sciences (Chinese Academy of Sciences). Female nude mice (4–5 weeks of age) were obtained from the animal department of the Institute of Biochemistry and Cell Biology, Chinese Academy of Sciences, Shanghai, and were maintained in pathogen-free conditions. Human acute leukemia cells CCRF-CEM or mouse myeloma cells SP2/0 were implanted in the armpit of 4–5-weeks-old female nude mice with the number of 1 × 10^7^ cells per mouse, which could grow into tumors by day 35. When the tumor size reached 500 mm^3^, all the mice received different treatments administered intraperitoneally. Based on in vitro optimization, inhibitor doses were as follows: the Akt isoform inhibitors plus DEX group received 2 × 10^−3^ µmol Akt isoform inhibitor and 0.1 mg DEX per mouse, the DEX group received 0.1 mg DEX per mouse, and the negative control group received only saline. Akt isoform inhibitors and DEX were administered on days 1–7 or days 1–11.

### Preparation of single-cell suspensions from tumors or spleen and flow cytometric analysis of cell apoptosis

Single-cell suspensions from tumors and spleen were prepared essentially by enzymatic digestion. Resected tumors and spleen were minced into small (1–2 mm^3^) pieces with a scalpel, and immersed in 10 ml of digestion mixture ((phosphate buffer saline) PBS, 0.5 mg/ml collagenase A, 0.2 mg/ml hyaluronidase and 0.02 mg/ml DNase I) per 0.25 g of tumor or spleen tissue. This mixture was incubated at 37 °C for 45 min on a rotating platform. The resulting cell suspensions were filtered sequentially through 60-and 40-µm cell strainers and washed with PBS. Then single-cell suspensions with cell concentration of 10^6^/ml were stained with FITC-labeled anti-annexin-V and/or prodium Iodide antibody (Sigma–Aldrich, USA), and analyzed by flow cytometry (BD Corp., USA).

### Statistical analyses

We performed statistical analysis using Student’s *t*-test. We considered results with *p* < 0.05 as statistically significant. Survival in animal experiments was represented with Kaplan–Meier curves and significance was estimated with the log-rank test.

## Electronic supplementary material

Supplementary Figure S1

Supplementary Figure S2

Supplementary Figure S3

Supplemental Experimental Procedures

Supplementary Figure Legends
