# Supplementary figures and images for: Akt2 mediates glucocorticoid resistance in lymphoid malignancies through FoxO3a/Bim axis and serves as a direct target for resistance reversal
Source: Cell Death Dis. 2019 Jan 1;9(10):1013. doi: 10.1038/s41419-018-1043-6 (PMC6312545; doi:10.1038/s41419-018-1043-6)

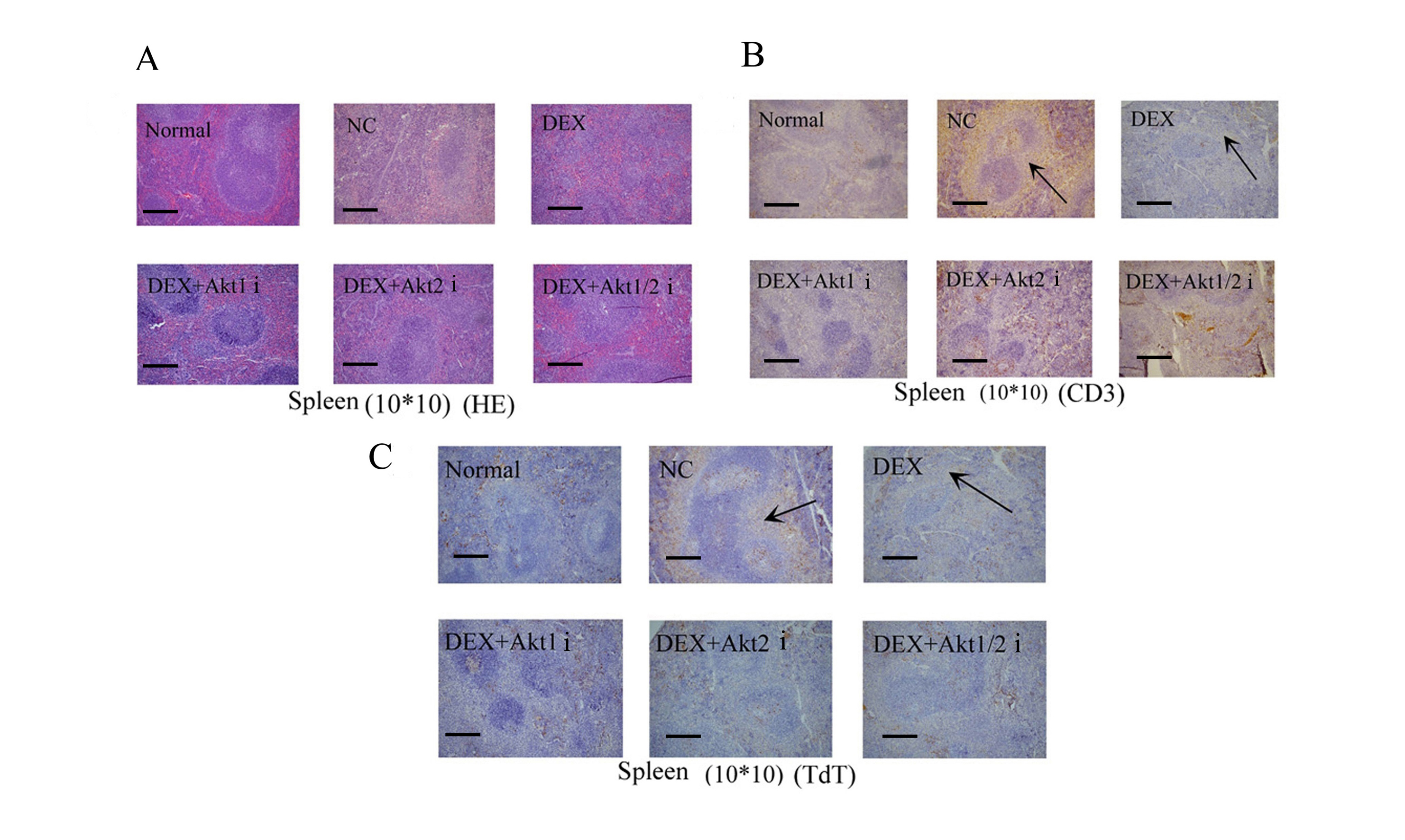

Supplement: Supplementary file 1 — Supplementary Figure S1 [file 41419_2018_1043_MOESM1_ESM.jpg]

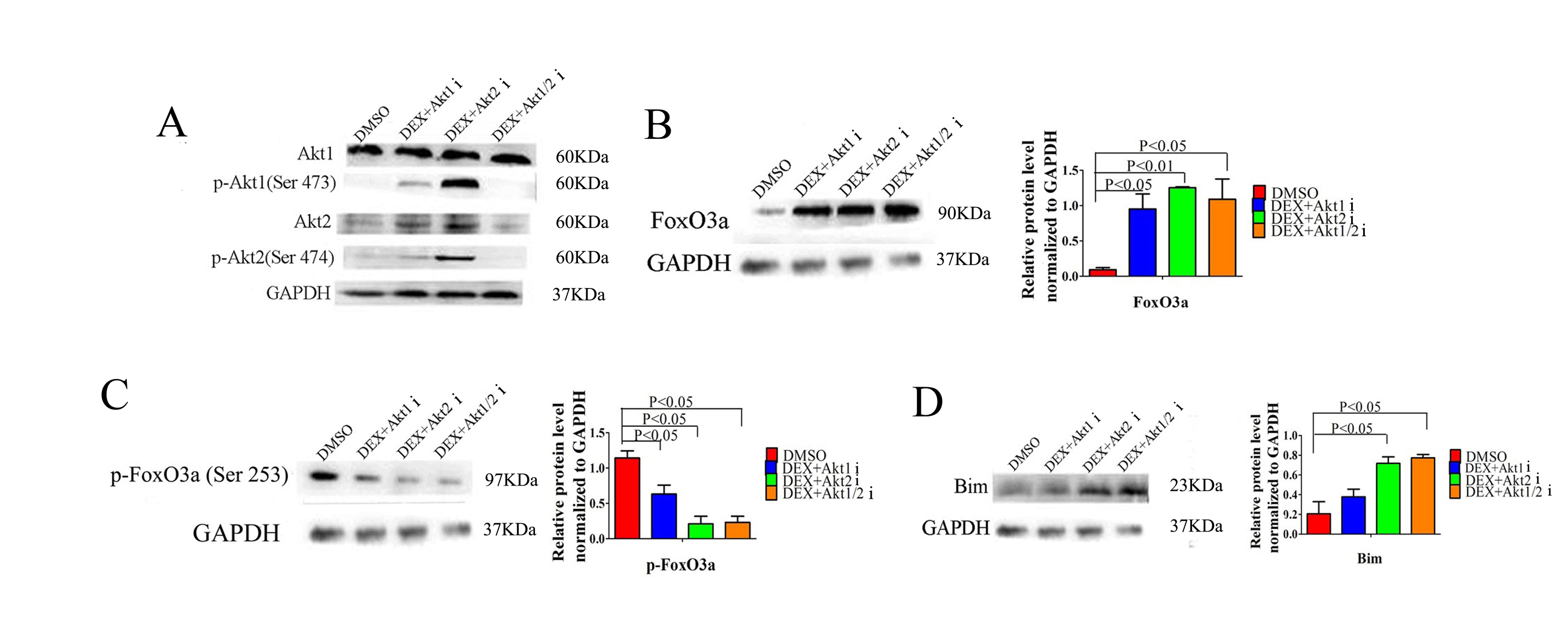

Supplement: Supplementary file 2 — Supplementary Figure S2 [file 41419_2018_1043_MOESM2_ESM.jpg]

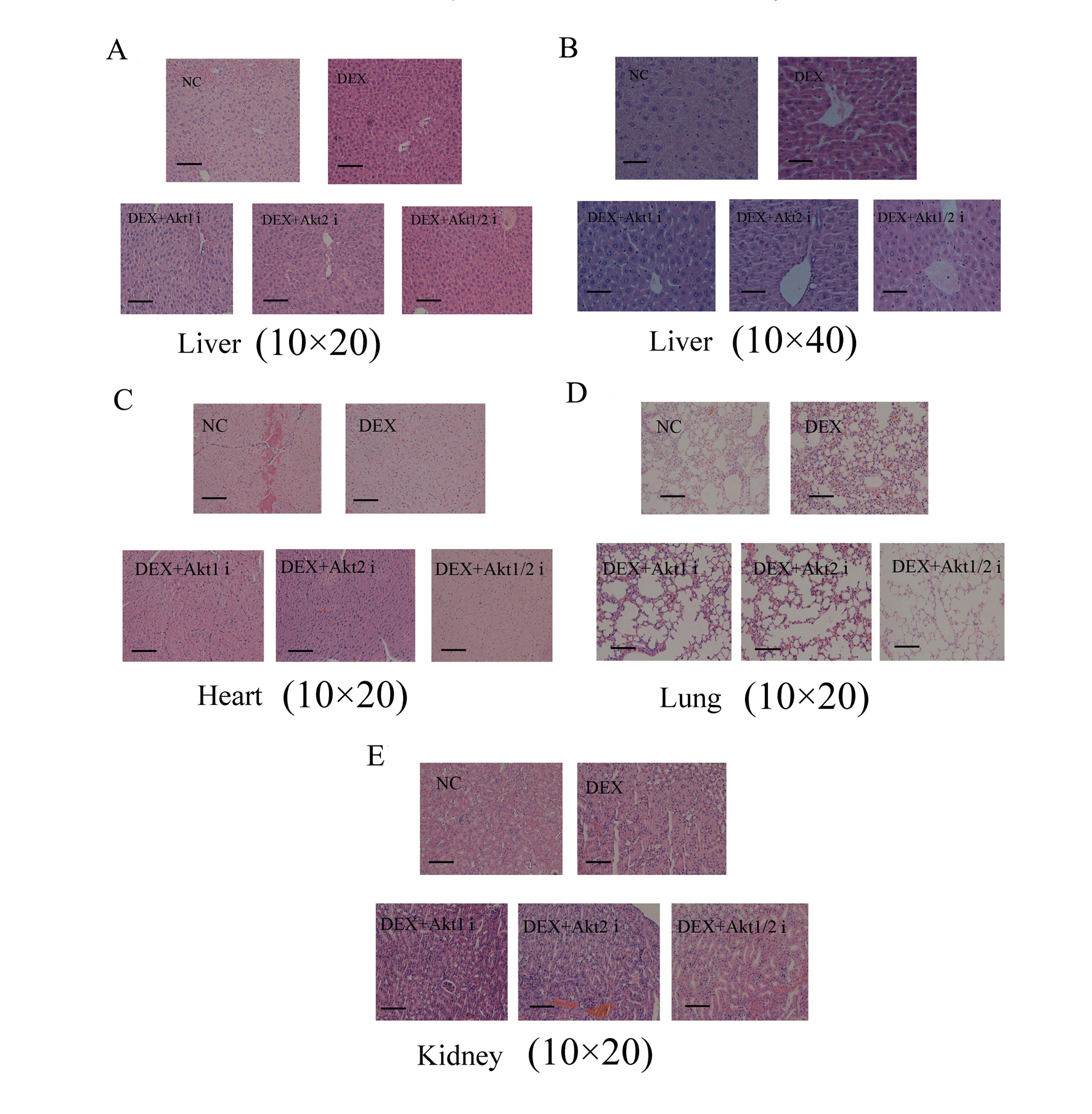

Supplement: Supplementary file 3 — Supplementary Figure S3 [file 41419_2018_1043_MOESM3_ESM.jpg]
