# Supplementary material for: Akt2 mediates glucocorticoid resistance in lymphoid malignancies through FoxO3a/Bim axis and serves as a direct target for resistance reversal
Source: Cell Death Dis. 2019 Jan 1;9(10):1013. doi: 10.1038/s41419-018-1043-6 (PMC6312545; doi:10.1038/s41419-018-1043-6)
Supplement: Supplementary file 4 — Supplemental Experimental Procedures [file 41419_2018_1043_MOESM4_ESM.docx]

**Supplemental Experimental Procedures**

**The preparation of GC-sensitizing agents**

Akt inhibitors were dissolved in DMSO at a concentration of 5 mM as a stock solution. LY294002 was dissolved in DMSO at a concentration of 50 mM as a stock solution. 2-DG was dissolved in water at a concentration of 0.3 M as a stock solution. GSK was dissolved in DMSO at a concentration of 100 mM as a stock solution. Dapt was dissolved in DMSO at a concentration of 20 mM as a stock solution. Stocks solutions were added directly to media to obtain the desired final concentrations.

**Cell culture**

The wild type lymphocytes, CCRF-CEM, Daudi, Raji, SP2/0, Molt-4 and Jurkat cells were cultured in RPMI 1640 medium (GIBCO, USA) containing 10% fetal bovine serum (FBS; Hyclone, USA). The L1210 cells and L-02 cells were cultured in DMEM (GIBCO, USA) containing 10% horse serum (GIBCO). All cells were incubated at 37°C in a 5% CO_2_ incubator.

**Construction of GC-resistant cell lines**

The drug concentration of inhibition to 50% of the ALL cells activity (IC50) was used as a measure of cellular drug resistance. The final drug concentrations ranged from 24 to 15000 µg/mL prednisolone or 1.5 to 800 µg/mL DEX for the GC-resistant cell lines and from 0.008 to 250 µg/mL prednisolone or 10^-5^ to 10^-2^ µg/mL DEX for GC-sensitive cell lines. Between this range was the GC intermediate resistant cell line. Human T-cell acute lymphoblastic leukemia CCRF-CEM cells were DEX intermediate resistant cells with the IC50 of DEX was 0.3 µM / 0.117 µg/mL. The GC-resistant CEM-DR cells were derived from the parental CCRF-CEM cells and cultured for 20 passages in the presence of 1 µM DEX to select for cells resistant to this agent. The CCRF-CEM and CEM-DR lines were routinely subcultured by 1:8 dilution of the cell suspension every 3–4 days to grow in the range 0.2–2 × 10^6^ cells per mL.

**Induction of apoptosis by DEX or by the combination of DEX and other GC-sensitizing agents**

DEX was dissolved in DMSO at a concentration of 25 mM as a stock solution or was dissolved in 100% ethanol at a concentration of 2.5 mM as a stock solution. DMSO without DEX or ethanol without DEX was used as the negative control. Apoptosis was analyzed several hours after treatment of DEX or other GC-sensitizing agents using an Annexin-V FITC Apoptosis Detection Kit (Sigma–Aldrich, USA). After an additional incubation of 48 h, the cells were harvested, stained with FITC-labeled anti-annexin-V and/or Prodium Iodide antibody, and analyzed by flow cytometry (BD Corp., USA).

**Cell viability assay**

Cell viability assays were performed with a Cell Counting Kit-8 (Dongren Corp.). Cells were plated in 24-well plates in triplicate at approximately 2~3 × 10^5^ cells per well and cultured in growth medium with different concentrations of DEX and Akt inhibitors. At the indicated time points, the numbers of cells per well were measured by the absorbance (450 nm) of reduced WST-8 (2-(2-methoxy-4-nitrophenyl)-3(4-nitrophenyl)-5-(2,4-isulfophenyl)-2H-tetrazolium,monoso-dium salt).

**Protein extraction and Western blot**

Cells were collected in SDS loading buffer (Sigma). Proteins were separated on 10% SDS-polyacrylamide gels and transferred to a PVDF membrane (Pall, USA). The membrane was then blocked with TBS/T solution containing 0.1% Tween 20, and 5% milk powder for 1 h at room temperature, followed by hybridization overnight at 4°C in TBS/T containing 0.1% Tween 20, 5% BSA powder and primary antibodies. Primary antibodies were detected by a peroxidase-coupled secondary antibody (Sigma) and chemiluminescence (Pierce), and quantified using Multi Gauge Image Analysis software (FUJIFILM). The following primary antibodies were used: rabbit anti-FoxO3a, p-FoxO3a, Akt, p-Akt, Akt1, Akt2, p-Akt1, and p-Akt2 (Cell Signaling Technology), anti-Bim (Sigma), GAPDH (Abcam) and β-actin (Abcam).

**Lymphocytes isolation, RNA isolation and quantitative polymerase chain reaction (PCR)**

Lymphocytes of bone marrow were isolated using Ficoll-Hypaque Solution according to the manufacturer’s instructions. Total RNA was isolated using Trizol Reagent (Invitrogen) according to the manufacturer’s instructions. Quantitative PCR was performed on unamplified total RNA using the NCodeTM quantitative PCR kit (Invitrogen) according to the manufacturer’s instructions. Akt mRNA was amplified using the sense primer 5’-GCACAAACGAGGGGAGTACAT-3’ and the antisense primer 5’-CCTCACGTTGGTCCACATC-3’. Akt1 mRNA was amplified using the sense primer 5’-GCTGCACGATAGCTTGGA-3’ and the antisense primer 5’-GATGACAGATAGCTGGTG-3’. Akt2 mRNA was amplified using the sense primer 5’-GGCCCCTGATCAGACTCTA -3’ and the antisense primer 5’-TCCTCAGTCGTGGAGGAGT -3’. The fold difference in the gene expressions of the samples was calculated using the equation –ΔCt. All reactions were performed in triplicate.
