# Supplementary material for: Akt2 mediates glucocorticoid resistance in lymphoid malignancies through FoxO3a/Bim axis and serves as a direct target for resistance reversal
Source: Cell Death Dis. 2019 Jan 1;9(10):1013. doi: 10.1038/s41419-018-1043-6 (PMC6312545; doi:10.1038/s41419-018-1043-6)
Supplement: Supplementary file 5 — Supplementary Figure Legends [file 41419_2018_1043_MOESM5_ESM.docx]

**Supplementary Figure Legends**

**Supplementary Figure S1. Treatment with DEX combined with Akt isoform inhibitors significantly reduces human leukemia cells highly expressing CD3 and TdT of spleens in tumor-bearing mice.** (A-C) Pathological histology of spleens with HE staining (G), CD3 staining (H) or TDT staining (I) in nude mice or tumor-bearing mice treated with saline (negative control, NC), DEX (0.1 mg), DEX plus Akt1 inhibitor (2×10^-3^ µmol), DEX plus Akt2 inhibitor (2×10^-3^ µmol) or DEX plus Akt1/2 inhibitor (2×10^-3^ µmol). Scale bar: 500 µm.

**Supplementary Figure S2. Treatment with DEX combined with Akt isoform inhibitors enhances the FoxO3a/Bim signaling pathway in liver cell.** (A) Western blot analysis of Akt1, p-Akt1 (Ser473), Akt2 and p-Akt2 (Ser474) in L-02 cells treated with DEX, DEX plus Akt1 inhibitor, DEX plus Akt2 inhibitor or DEX plus Akt1/2 inhibitor. (B-D) Western blot analysis of FoxO3a/Bim signaling proteins in L-02 cells treated with DEX, DEX plus Akt1 inhibitor, DEX plus Akt2 inhibitor or DEX plus Akt1/2 inhibitor. Bar graphs in panels B-D represent mean ± SD.

**Supplementary Figure S3. The influence on pathological histology of vital organs by Akt isoform inhibitors *in vivo*** (A-E) Pathological histology of liver, heart, lung or kidney with HE staining when mice were treated with saline, DEX, DEX plus each of Akt isoform inhibitors. Scale bar: 250 µm for A, C, D, E; 100µm for B.
